# Highly suspected fulminant myocarditis induced by the immune checkpoint inhibitor tislelizumab: a case report with third-degree atrioventricular block and recurrent ventricular tachycardia

**DOI:** 10.3389/fcvm.2026.1726826

**Published:** 2026-02-13

**Authors:** Junru Qu, Miao Liu, Neil Johnson, Wenwen Zhang, Zhiqiang Wang, Yingyi Su, Beibei Du, Yuquan He

**Affiliations:** 1 Department of Cardiology, China-Japan Union Hospital of Jilin University, Changchun, China; 2 Changchun University of Chinese Medicine, Changchun, China

**Keywords:** immune checkpoint inhibitors, immune-related adverse events, tislelizumab, intrahepatic cholangiocarcinoma, myocarditis, third-degree atrioventricular block, case report

## Abstract

**Introduction:**

Immune checkpoint inhibitors (ICIs) have significantly advanced the treatment of malignant tumors. Tislelizumab, a recombinant humanized anti–programmed death 1 (PD-1) monoclonal antibody, is widely used in the management of advanced cancers. However, like other ICIs, tislelizumab can trigger immune-related adverse events (irAEs). Among irAEs, immune checkpoint inhibitor-associated myocarditis (ICI-associated myocarditis) is rare but life-threatening, underscoring the need for early recognition and intervention. This case report describes a case of highly suspected fulminant myocarditis induced by ICI in a patient with gallbladder adenocarcinoma and intrahepatic cholangiocarcinoma (iCCA) following treatment with tislelizumab.

**Case presentation:**

A 72-year-old female presented with a 4-h history of palpitations and dyspnea on June 16, 2025. Initial electrocardiography (ECG) revealed ventricular tachycardia (VT), which was successfully terminated with 200 J cardioversion. Subsequent monitoring showed third-degree atrioventricular block (AVB). She had been diagnosed with iCCA and gallbladder adenocarcinoma on May 7, 2025, and had undergone partial hepatectomy and cholecystectomy. She later received intravenous tislelizumab (200 mg) on May 22, 2025, followed by a second 200 mg dose on June 10, with no adverse effects observed during either infusion. Upon admission, investigations revealed no coronary abnormalities on coronary computed tomography, a left ventricular ejection fraction (LVEF) of 42%, markedly elevated cardiac biomarkers [e.g., cardiac troponin I (cTnI) 3.80 μg/L], hypoxemia, and 24-h Holter monitoring that documented short episodes of VT and third-degree AVB. Based on the temporal relationship to ICI therapy, the clinical presentation, laboratory and imaging findings, and the exclusion of alternative etiologies, fulminant ICI-associated myocarditis was highly suspected. Treatment included noninvasive mechanical ventilation, temporary pacing, high-dose methylprednisolone, intravenous immunoglobulin, antiarrhythmic agents, and heart failure (HF) management. On hospital day 4, the patient's symptoms had improved, although third-degree AVB persisted. On hospital day 15, her atrioventricular conduction had improved from third-degree to first-degree block, with cardiac biomarkers decreasing to much lower levels. On hospital day 18, repeat transthoracic echocardiography (TTE) showed an LVEF of 49% with generalized hypokinesis of the left ventricular wall, and a repeat ECG revealed first-degree AVB, complete right bundle branch block, and left anterior fascicular block. She was discharged on the same day. During the subsequent 2-month follow-up after discharge, the patient remained clinically stable but developed symptoms of muscle weakness, for which she did not seek further medical evaluation.

**Conclusion:**

ICI-associated myocarditis is characterized by a low incidence but carries a high risk of fatal outcomes. Early recognition of severe cases and timely intervention can substantially improve prognosis. In this case, fulminant myocarditis related to the ICI tislelizumab was highly suspected but not pathologically confirmed, as cardiac magnetic resonance imaging and endomyocardial biopsy were not performed. Despite this limitation, the case may still provide a useful clinical reference for the diagnosis and management of severe ICI-associated myocarditis.

## 1 Introduction

In the past decade, immune checkpoint inhibitors (ICIs) have significantly transformed the treatment of malignant tumors by counteracting tumor immune evasion mechanisms (1). However, with the broader use of ICIs, immune-related adverse events (irAEs) have attracted growing clinical attention. Among these, immune checkpoint inhibitor-associated myocarditis (ICI-associated myocarditis) is rare but carries a high mortality, underscoring the urgent need for early detection and timely intervention (2). However, clinical research on ICI-associated myocarditis, particularly regarding the management and outcomes of severe cases, remains limited. This article presents a case of fulminant myocarditis in a patient with gallbladder adenocarcinoma and intrahepatic cholangiocarcinoma (iCCA), with the myocarditis developing after treatment with tislelizumab—an anti–programmed death 1 (PD-1) monoclonal antibody and a type of ICI—and considered highly likely to be ICI related. The core features of this case included third-degree atrioventricular block (AVB), recurrent ventricular tachycardia (VT), acute heart failure (HF), and markedly elevated cardiac biomarkers. Through a combination of high-dose corticosteroid pulse therapy, temporary pacemaker implantation, heart failure management, and antiarrhythmic therapy, the patient ultimately achieved a relatively favorable outcome.

## 2 Case presentation

**Figure 1:**
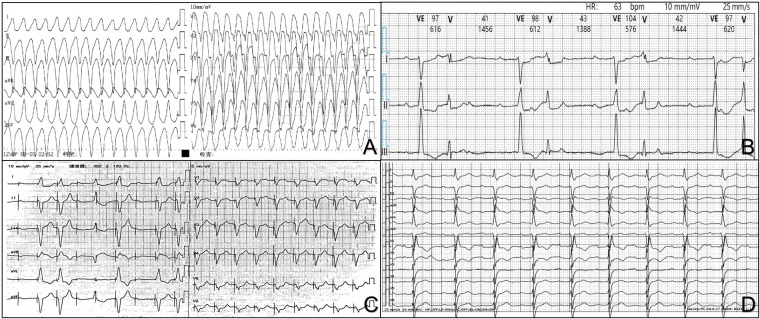
ECG changes in the patient with ICI-associated myocarditis: Emergency ECG on June 16, 2025 shows VT with a wide QRS complex morphology and a rapid ventricular rate. **(A)** Holter monitoring during hospitalization displays third-degree AVB, and frequent multiform ventricular premature beats. **(B)** After temporary pacemaker implantation, the ECG shows paced rhythms with a pacing rate of 110 bpm to suppress VT. **(C)** Follow-up ECG on hospital day 18 reveals first-degree AVB, newly developed complete right bundle branch block, and left anterior fascicular block representing the residual conduction system damage despite clinical improvement. **(D)** (AVB, atrioventricular block; ICI-associated myocarditis, immune checkpoint inhibitor-associated myocarditis; ECG, electrocardiography; VT, ventricular tachycardia).

A 72-year-old female presented to the Emergency Department of China-Japan Union Hospital of Jilin University on June 16, 2025, with a 4-hour history of palpitations and dyspnea—symptoms that prompted urgent medical evaluation. Electrocardiography (ECG) revealed VT (Figure 1A), with a ventricular rate of 209 beats per minute (bpm) and a blood pressure of 92/56 mmHg, indicating hemodynamic instability. Synchronized direct current cardioversion (200 J) was performed immediately, successfully terminating the VT; subsequent ECG monitoring revealed third-degree AVB and frequent premature ventricular beats. The patient was then admitted to our department.

On admission, her past medical history was as follows: she had been diagnosed with moderately to poorly differentiated iCCA and gallbladder adenocarcinoma on May 7, 2025, and shortly thereafter underwent partial hepatectomy and cholecystectomy. During her previous surgical hospitalization, a preoperative ECG on May 5 demonstrated first-degree AVB (Supplementary Figure S1), and transthoracic echocardiography (TTE) on May 6 showed a left ventricular ejection fraction (LVEF) of 60%. At that time, cardiac biomarkers including cardiac troponin I (cTnI), myoglobin, creatine kinase–MB (CK-MB), and N-terminal pro–B-type natriuretic peptide (NT-proBNP) were within normal reference ranges. The patient had no history of diabetes mellitus, coronary artery disease, autoimmune disorders, or other relevant comorbidities. As systemic therapy for her liver and gallbladder malignancies, she received intravenous tislelizumab at a dose of 200 mg on May 22 and a second 200 mg dose on June 10, with no adverse reactions or discomfort reported during either infusion. On admission, her vital signs were as follows: temperature 36.6℃, heart rate 59 bpm, respiratory rate 25 breaths per minute, and blood pressure 110/82 mmHg. Pulmonary auscultation revealed coarse breath sounds with scattered fine moist crackles in both lung fields, and the cardiac rhythm was irregular. Investigations on admission showed the following results: coronary computed tomography revealed no obvious abnormalities; TTE demonstrated an LVEF of 42%, representing a marked decline from the LVEF of 60% measured on May 6, prior to initiation of tislelizumab. In addition, global longitudinal strain (GLS), derived from speckle-tracking imaging (STI), was severely reduced to −5% (normal reference range: −15% to −20%), characterized by a pattern of diffuse longitudinal strain reduction. Cardiac biomarkers were markedly elevated: NT-proBNP 2,480 μg/L (reference range 300–900 μg/L), cTnI 3.80 μg/L (reference range 0.01–0.02 μg/L), myoglobin >900.00 μg/L (reference range 23–112 μg/L), and CK-MB 170 μg/L (reference range 2–11 μg/L). Liver function tests were abnormal, with elevated alanine aminotransferase (ALT) 88.40 U/L, aspartate aminotransferase (AST) 181.56 U/L, and lactate dehydrogenase (LDH) 922.21 U/L. Arterial blood gas analysis demonstrated hypoxemia, with a partial pressure of oxygen (PaO_2_) of 49.8 mmHg and oxygen saturation (SaO2) of 81%. Serum electrolytes were within normal limits: K^+^ 3.80 mmol/L, Ca^2+^ 2.25 mmol/L, and Mg^2+^ 0.96 mmol/L. Elevated inflammatory markers were also noted, including high-sensitivity C-reactive protein (hs-CRP) at 82.43 mg/L (reference range <10 mg/L) and an erythrocyte sedimentation rate (ESR), assessed by the Westergren method, of 69 mm/h (clinical reference range for women >50 years of age 0–30 mm/h), indicating active systemic inflammation. During hospitalization, 24-h Holter monitoring revealed short episodes of VT, third-degree AVB, and frequent multiform premature ventricular beats (Figure 1B). Key clinical events during hospitalization are summarized in Table 1. Notably, cardiac magnetic resonance (CMR) imaging and endomyocardial biopsy (EMB)—the gold standard for the diagnosis of myocarditis—were not performed, primarily because of the patient's unstable clinical condition.

**Table 1:** Timeline of our case.

Timeline	
40 days prior to presentation	• The patient was diagnosed with gallbladder adenocarcinoma and iCCA and subsequently underwent partial hepatectomy and cholecystectomy
25 days prior to presentation	• The patient received the first dose of tislelizumab (200 mg in 20 mL) without obvious discomfort
6 days prior to presentation	• The patient received the second dose of tislelizumab (200 mg in 20 mL) without obvious discomfort
4 h prior to presentation	• The patient developed persistent palpitations accompanied by dyspnea
At presentation	• The patient presented with persistent VT (HR 202 bpm, BP 92/56 mmHg). Electrical cardioversion was performed immediately, successfully terminating the VT
	• Third-degree AVB was detected after cardioversion
	• Cardiac biomarkers were markedly elevated (NT-proBNP 2,480 μg/L, cTnI 3.80 μg/L, myoglobin >900 μg/L, CK-MB 170 μg/L), and the LVEF was 42%, while GLS was severely reduced to −5%
	• Noninvasive mechanical ventilation was initiated
	• A temporary transvenous pacemaker was implanted, and the pacing rate was set at 110 bpm
	• Antiarrhythmic therapy was initiated with intravenous magnesium and potassium magnesium aspartate
	• Heart failure therapy was initiated with intravenous recombinant human brain natriuretic peptide and torasemide, along with oral sacubitril/valsartan and vericiguat
	• Immunosuppressive therapy was started with high-dose corticosteroid pulse therapy (intravenous methylprednisolone 1 g once daily for 3 days, followed by 0.5 g once daily with gradual tapering) and intravenous immunoglobulin 20 g once daily for 4 consecutive days
Hospital day 2	• As the patient's dyspnea worsened and chest ultrasonography revealed a left-sided pleural effusion approximately 7 cm in depth, therapeutic thoracentesis with drainage was performed
Hospital day 3	• The dose of methylprednisolone was reduced to 0.5 g by intravenous infusion once daily, with subsequent gradual tapering
Hospital day 4	• The patient's symptoms improved, and the temporary pacing rate was reduced to 70 bpm
Hospital day 15	• Intrinsic atrioventricular conduction recovered, and the temporary pacemaker was removed
Hospital day 18	• Myocardial markers decreasing to stable levels, the patient discharged with improvement
Approximately 60 days after presentation	• The patient remained clinically stable but developed symptoms of muscle weakness

AVB, atrioventricular block; BP, blood pressure; bpm, beats per minute; CK-MB, creatine kinase–MB; cTnI, cardiac troponin I; ECG, electrocardiography; HR, heart rate; iCCA, intrahepatic cholangiocarcinoma; LVEF, left ventricular ejection fraction; NT-proBNP, N-terminal pro–B-type natriuretic peptide; VT, ventricular tachycardia.

Serologic testing for viral antibodies was negative, and a normal coronary computed tomography scan effectively ruled out acute coronary syndrome (ACS) as an alternative cause of myocardial injury. Further evaluation excluded other potential etiologies of myocardial involvement. A chest x-ray performed on admission and a lung computed tomography scan obtained on April 25, 2025, showed no hilar or paratracheal lymphadenopathy and no radiologic features suggestive of pulmonary sarcoidosis, thereby allowing cardiac sarcoidosis to be excluded. Stress-induced cardiomyopathy (Takotsubo syndrome) was considered unlikely, as no emotional or physical stressors were identified before symptom onset and the patient had markedly elevated troponin levels. Cardiac amyloidosis was also deemed unlikely because there was no evidence of “GLS apical sparing” (the patient instead exhibited diffuse longitudinal strain abnormalities), no systemic organ involvement (e.g., renal impairment, peripheral neuropathy), and markedly elevated troponin levels, whereas in cardiac amyloidosis troponin is typically only mildly elevated or within the normal range.

Taken together, these features—the recent exposure to tislelizumab with a clear temporal relationship to symptom onset; the marked elevation of cardiac biomarkers; the presence of sustained VT, newly developed third-degree AVB, and left ventricular systolic dysfunction with severely reduced GLS; and the systematic exclusion of alternative causes of myocardial injury, including ACS, viral myocarditis, cardiac sarcoidosis, stress-induced (Takotsubo) cardiomyopathy, and cardiac amyloidosis—were most consistent with a working diagnosis of highly suspected fulminant ICI-associated myocarditis induced by tislelizumab. Accordingly, the case was managed as highly suspected fulminant ICI-associated myocarditis complicating tislelizumab therapy.

Upon admission, noninvasive mechanical ventilation was initiated. In view of the third-degree AVB and paroxysmal VT documented on Holter monitoring, a temporary transvenous pacemaker was implanted, with the pacing rate set at 110 beats/min to suppress VT by overdrive pacing (Figure 1C). To attenuate the inflammatory response of myocarditis, high-dose corticosteroid pulse therapy was started: methylprednisolone 1 g was administered intravenously once daily for 3 days, then reduced to 0.5 g intravenously once daily with subsequent gradual tapering, and finally transitioned to oral methylprednisolone 40 mg once daily; in addition, intravenous immunoglobulin 20 g was administered once daily for 4 consecutive days. Concurrently, intravenous potassium magnesium aspartate and amiodarone were given for arrhythmia control. For HF management, intravenous recombinant human brain natriuretic peptide and torsemide were administered, together with oral sacubitril/valsartan and vericiguat. Additionally, intravenous compound glycyrrhizin was given for hepatoprotection.

On hospital day 2, the patient's dyspnea worsened and she developed orthopnea. Bedside thoracic ultrasonography revealed a large left-sided pleural effusion with a maximal thickness of approximately 7 cm, which was drained by therapeutic thoracentesis. We recommended CMR imaging and EMB to further confirm the diagnosis, as well as implantation of an implantable cardioverter-defibrillator (ICD) for secondary prevention of malignant ventricular arrhythmias. However, in view of the anticipated poor prognosis and financial constraints, both EMB and ICD were temporarily declined. On hospital day 4, her symptoms had improved; however, third-degree AVB persisted, and the temporary pacemaker rate was ultimately reduced to 70 beats/min.

**Figure 2:**
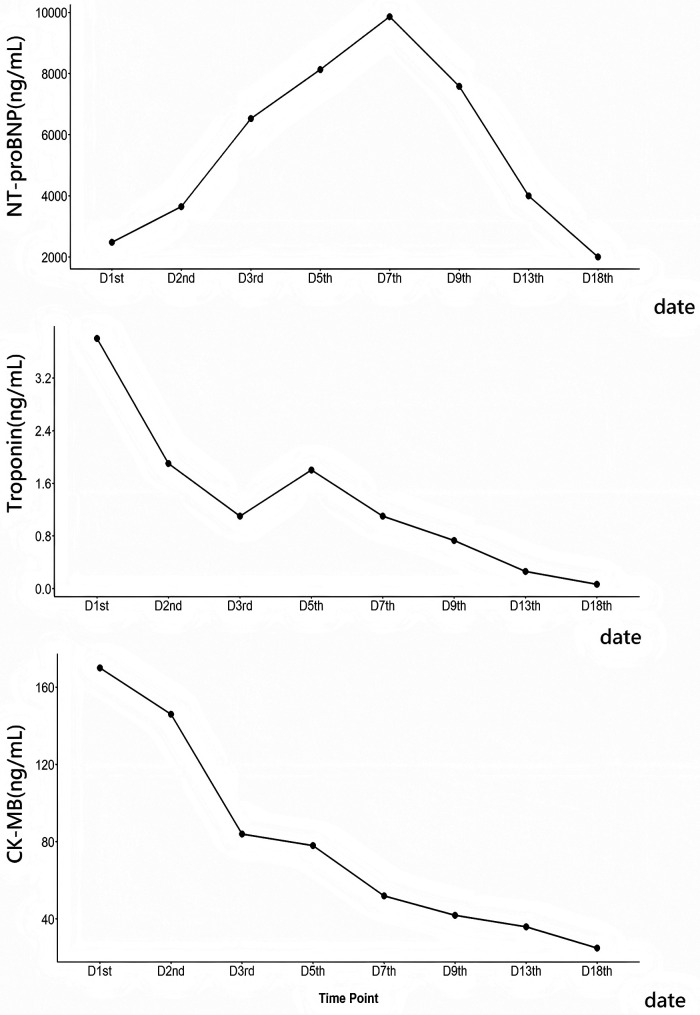
Timeline of NT-proBNP, CK-MB, and Troponin I levels during hospitalization. (NT-proBNP, N-terminal pro-B-type natriuretic peptide; CK-MB, Creatine kinase-MB).

On hospital day 15, her atrioventricular conduction had improved from third-degree to first-degree block. The temporary pacemaker was removed, and subsequent Holter monitoring revealed first-degree AVB, complete right bundle branch block, and left anterior fascicular block. On hospital day 18, the patient's symptoms had substantially resolved, cardiac biomarkers had decreased to stable levels (Figure 2), and follow-up TTE showed an LVEF of 49% with generalized hypokinesis of the left ventricular wall, whereas a follow-up ECG demonstrated first-degree AVB, complete right bundle branch block, left anterior fascicular block, and a ventricular rate of 76 beats/min (Figure 1D). During 2 months of follow-up, the patient remained clinically stable, but developed symptoms of muscle weakness and did not seek further medical evaluation.

## 3 Discussion

This article describes a case of highly suspected fulminant myocarditis occurring 29 days after the first administration of tislelizumab, characterized by recurrent VT, third-degree AVB, and HF. After 16 days of comprehensive treatment, her clinical condition gradually stabilized and improved.

ICIs have revolutionized cancer treatment. Three major classes of ICIs are currently used in clinical practice: PD-1 inhibitors, programmed death-ligand 1 (PD-L1) inhibitors, and cytotoxic T-lymphocyte-associated antigen 4 (CTLA-4) inhibitors. PD-1 antibodies block the interaction between PD-1 and PD-L1, restore the function of effector CD8+ T cells, and enable these cells to regain the ability to recognize, and exert cytotoxicity against cancer cells, thereby effectively prolonging the overall survival of patients with advanced malignancies (3).

However, among patients receiving anti-PD-1 antibodies, the incidence of irAEs of any grade is approximately 66.4%–75.1%, and the incidence of severe irAEs ranges from 6% to 19.8% (4). ICI-associated myocarditis, a form of irAEs, has a relatively low incidence, estimated at approximately 1.14%. Despite its low frequency, it is characterized by acute onset and rapid progression, with a median time to onset of 34 days after initiation of ICI therapy, and up to 46% of affected patients experiencing major adverse cardiovascular events (MACE) (5). Excessive T-cell activation, proinflammatory cytokine storm, and aberrant activation of the JAK-STAT signaling pathway are thought to represent key mechanisms underlying ICI-associated myocarditis, which, to some extent, may explains the efficacy of combination therapies targeting these pathways (glucocorticoids, abatacept, and ruxolitinib) (6). High-risk factors for ICI-associated myocarditis include combined ICI therapy (e.g., CTLA-4 inhibitor plus PD-1/PD-L1 inhibitor), concurrent irAEs such as myositis or myasthenia gravis, and preexisting cardiovascular comorbidities. In addition, the presence of high-risk clinical features—such as marked elevation of cardiac biomarkers, particularly troponin; early malignant arrhythmias; and hemodynamic instability—helps identify patients at risk of severe myocarditis and poor prognosis (7). In our patient, advanced age, pre-existing first-degree AVB, and the subsequent development of marked troponin elevation, sustained VT, and hemodynamic instability together indicate a high-risk profile and are consistent with a fulminant presentation.

The clinical manifestations of ICI-associated myocarditis are highly heterogeneous and may include chest pain and dyspnea; acute HF and various arrhythmias; elevation of cardiac biomarkers, particularly troponin; and abnormal ECG findings such as ST-T changes, prolonged QRS duration, and prolonged QT interval (8). A multicenter retrospective registry study developed a risk score for ICI-associated myocarditis and identified that active thymoma, incremental elevations in cardiac troponin (20–2000-fold above the upper reference limit), low Sokolow-Lyon QRS voltage on ECG (≤0.5 mV), LVEF <50%, and the presence of cardiomuscular symptoms were independent predictors of major cardiomyotoxic events within 30 days (9).

After ruling out other causes of myocardial injury (e.g., ACS, infectious myocarditis), a diagnosis of ICI-associated myocarditis can be considered on the basis of the patient's treatment history, newly developed symptoms, and dynamic changes in cardiac biomarkers. CMR imaging is recommended as a core component of the diagnostic workup for ICI-associated myocarditis, and EMB remains the gold standard for pathological confirmation. Regrettably, we were unable to perform these two key examinations in this patient, which undoubtedly represents the most notable limitation of this case. In the absence of CMR and EMB, we emphasize that the diagnosis in this case should be regarded as clinical and probability-based rather than pathologically confirmed ICI-associated myocarditis. Despite this limitation, a diagnosis of ICI-associated myocarditis in our patient is still clinically reasonable, based on the following key evidence: a clear history of ICI exposure with a temporal relationship to symptom onset; markedly elevated cTnI and CK-MB levels and increased inflammatory markers; a clinical syndrome characterized by sustained VT and newly developed third-degree AVB; a LVEF of 42%; and a markedly reduced GLS of −5% with a pattern of diffuse, rather than segmental, reduction in longitudinal strain. Furthermore, other potential etiologies of myocardial involvement—such as ACS, infectious myocarditis, cardiac sarcoidosis, Takotsubo syndrome, and cardiac amyloidosis—were systematically excluded. The favorable response to treatment (i.e., resolution of symptoms, reduction in cardiac biomarkers, improvement in LVEF to 49%, and recovery of AVB from third-degree to first-degree) provides additional retrospective support for the validity of both our diagnostic assessment and our therapeutic decisions.

Once ICI-associated myocarditis is suspected or diagnosed, risk stratification should be performed. Fulminant myocarditis is defined by the presence of hemodynamic instability, HF requiring noninvasive or invasive ventilatory support, advanced AVB, or severe ventricular arrhythmias (10).

In our patient, the initial hemodynamic compromise (blood pressure 92/56 mmHg), the need for noninvasive mechanical ventilation, sustained VT requiring immediate cardioversion, and newly developed third-degree AVB fulfilled these criteria, supporting classification as a fulminant presentation of highly suspected ICI-associated myocarditis.

Arrhythmias induced by ICIs are often accompanied by myocarditis and can present in various forms; among these, advanced AVB, VT, and ventricular fibrillation (VF) are the most life-threatening (11). A retrospective multicenter study found that approximately 15% of patients with ICI-associated myocarditis experienced at least one life-threatening ventricular arrhythmia, with sustained VT occurring in 10.9% of all patients (12). When ventricular arrhythmias (e.g., sustained VT) lead to hemodynamic instability, immediate electrical cardioversion is indicated (13). In patients who have experienced severe ventricular arrhythmias, implantation of an ICD may be considered for secondary prevention; however, multiple factors—including the patient's expected survival time, the activity of myocardial inflammation, the potential for recovery of cardiac function, and the persistence of arrhythmia risk—should be taken into account (14).

AVB is a relatively characteristic feature of ICI-associated myocarditis, and complete AVB is the most common form, with its occurrence being associated with poor prognosis (12). A prolonged PR interval (>200 ms) on ECG prior to the onset of AVB and a lower LVEF may serve as predictive factors for the development of AVB. The temporal pattern of AVB in ICI-associated myocarditis is heterogeneous. Although some cases show gradual progression, a multicenter study reported that, among 34 patients with ICI-associated myocarditis, 7 developed third-degree AVB, all occurring 21–33 days after the first ICI dose, and that all patients with complete AVB had markedly elevated cardiac biomarkers (15). Our patient developed third-degree AVB 29 days after the first tislelizumab infusion, with an abrupt onset. This acute conduction failure may be mechanistically related to rapid immune-mediated infiltration and injury of the cardiac conduction system during severe acute myocardial inflammation. The markedly elevated cardiac biomarkers in our patient (cTnI 3.80 μg/L, myoglobin >900 μg/L, and CK-MB 170 μg/L) indicate extensive and severe myocardial injury, consistent with diffuse cardiac damage that likely also involves the conduction system.

For patients with severe AVB, timely pacing therapy is of paramount importance because severe bradycardia can lead to hemodynamic compromise, myocardial ischemia, syncope, and malignant ventricular arrhythmias. Therefore, temporary pacing, or even permanent pacing, should be considered when necessary. A meta-analysis has shown that pacing therapy reduces mortality in patients with ICI-associated myocarditis complicated by severe AVB (16). Selection of an appropriate pacing strategy requires careful consideration. Case reports have shown that in some patients with third-degree AVB, atrioventricular conduction can recover within a short period after aggressive immunosuppressive treatment, whereas in others it may take a long time to recover or may not recover at all (17, 18). However, the definition of “restoration” of atrioventricular conduction in such patients has not yet been clearly or uniformly established, and their overall survival and prognosis are generally poor. In our patient, third-degree AVB was present, for which a temporary pacemaker was promptly implanted and the pacing rate was increased to 110 beats/min to suppress VT, a crucial step in her management. On hospital day 15, atrioventricular conduction had improved from third-degree AVB to first-degree AVB, with new complete right bundle branch block and left anterior fascicular block.

For patients with severe ICI-associated myocarditis, early identification and intervention are particularly critical. Itzhaki Ben Zadok et al. found that the 1-year cardiovascular mortality of patients with ICI-associated myocarditis was lower than previously reported and was mostly confined to those with a severe presentation (29% vs. 5% in nonsevere cases). This improvement may be attributed to increased awareness of irAEs and advances in treatment (19). Meanwhile, Pereyra Pietri et al. reported that the first 120 days following diagnosis constitute the period of highest risk for adverse cardiac events in patients with ICI-associated myocarditis; beyond this time window, their risk does not differ significantly from that of patients treated with ICIs who did not develop myocarditis (20). Therefore, early recognition of severe cases and timely intervention may help these patients safely traverse this “critical period,” thereby significantly reducing the mortality of ICI-associated myocarditis.

## 4 Conclusion

ICI-associated myocarditis is characterized by a low incidence but carries a high risk of fatal outcomes. Marked elevation of cardiac biomarkers and the presence of malignant ventricular arrhythmias constitute key indicators for risk stratification. Early recognition of severe cases and timely intervention can substantially improve prognosis. In this case, fulminant myocarditis related to tislelizumab was highly suspected but not pathologically confirmed, as CMR imaging and EMB were not performed. Despite this limitation, the case may still provide a useful clinical reference for the diagnosis and management of severe ICI-associated myocarditis.

## Data Availability

The original contributions presented in the study are included in the article/Supplementary Material, further inquiries can be directed to the corresponding authors.

## Supplementary material

Supplementary Figure S1 Prior to surgery on May 7, 2025, first-degree AVB was observed on ECG, with a PR interval of approximately 260 ms.
