# Supplementary figures and images for: Highly suspected fulminant myocarditis induced by the immune checkpoint inhibitor tislelizumab: a case report with third-degree atrioventricular block and recurrent ventricular tachycardia
Source: Front Cardiovasc Med. 2026 Feb 13;13:1726826. doi: 10.3389/fcvm.2026.1726826 (PMC12946105; doi:10.3389/fcvm.2026.1726826)

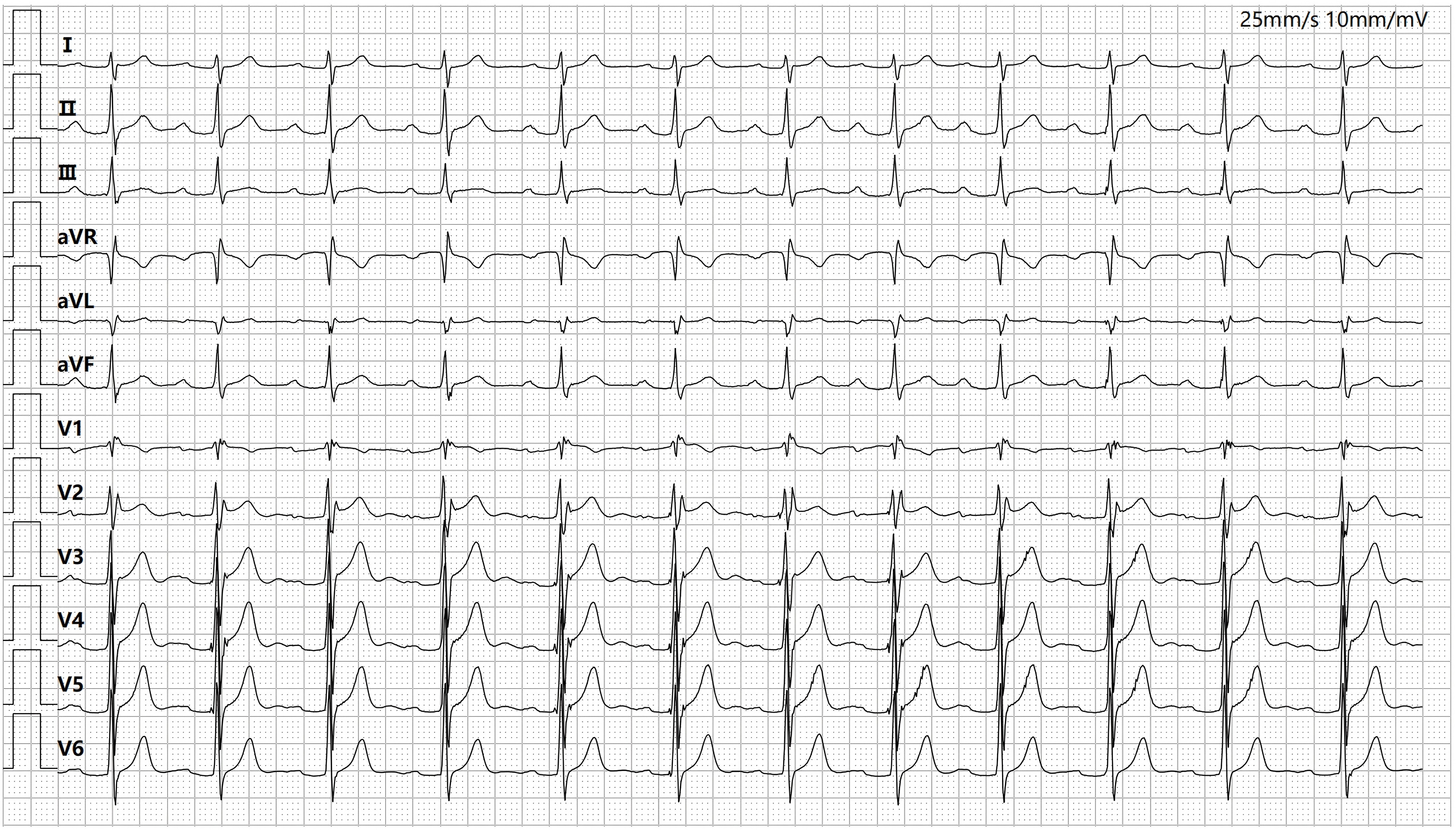

Supplement: Supplementary Figure S1 — Prior to surgery on May 7, 2025, first-degree AVB was observed on ECG, with a PR interval of approximately 260 ms. [file Image1.jpeg]
